# Ubiquitination regulation in coronary microcirculation dysfunction: focus on the endothelial cells

**DOI:** 10.3389/fcvm.2026.1952434

**Published:** 2026-10-06

**Authors:** Qingying Zhang, Tingting Wang, Siyu Wang, Yushan Chen, Yiwen Sheng, Haodi Zhang, Yunhao Wang, Boyuan Jin, Chengjun Hua, Xinyi Han

**Affiliations:** 1 Heart Center/National Regional (Traditional Chinese Medicine) Cardiovascular Diagnosis and Treatment Center, The First Affiliated Hospital of Henan University of Traditional Chinese Medicine, Zhengzhou, Henan, China; 2 Department of Cardiology, The First Affiliated Hospital of Henan University of Traditional Chinese Medicine, Zhengzhou, Henan, China; 3 The First Clinical Medical College, Henan University of Traditional Chinese Medicine, Zhengzhou, Henan, China

**Keywords:** coronary microcirculation dysfunction, deubiquitination, endothelial cells (ECs), eNOS, inflammation, ubiquitination

## Abstract

Basic and clinical research on coronary microvascular disease (CMD) has spanned half a century. As a pathogenesis independent of epicardial coronary stenosis, CMD has become a major hotspot in cardiovascular medicine. Ubiquitination, a vital post-translational modification in eukaryotic cells, precisely regulates target proteins via the E1-E2-E3 enzymatic cascade and plays a critical role in cardiovascular homeostasis and pathophysiology. Recent evidence indicates that the ubiquitin-proteasome system (UPS) is deeply involved in the development of CMD by modulating nitric oxide production, barrier function, and inflammatory signaling in coronary microvascular endothelial cells. This review summarizes recent advances in ubiquitination, elaborates its molecular mechanisms in regulating coronary microcirculation, and dissects its pathogenic roles in diabetes, hypertension, and myocardial ischemia/reperfusion injury (MIRI).We further discuss potential therapeutic strategies and translational challenges of targeting the ubiquitination pathway for CMD, including proteasome inhibitors, E3 ligase modulators, deubiquitinase agonists, and PROTACs, aiming to provide new insights for future research.

## 1 Introduction

Coronary microvascular dysfunction (CMD) is one of the important etiologies of ischemic heart disease, and coronary microvascular endothelial cells (CMECs) play a central role in maintaining coronary blood flow and the balance of myocardial metabolites (1, 2). Coronary microvascular endothelial cells are a key component in maintaining the structural integrity and normal function of the coronary microcirculation (3), and their dysfunction is closely associated with a variety of cardiovascular diseases, such as hypertension, diabetic myocardial injury and ischemia-reperfusion injury (MIRI) (4, 5). Ubiquitination affects the proliferation, inflammatory response, cell junctions, and barrier function of CMECs by regulating protein degradation, activity, and localization (6, 7) (Table 1). Understanding the regulation of endothelial cell function by the ubiquitination system is of great significance for comprehending and treating cardiovascular diseases.

**Table 1:** Effects of ubiquitination on endothelial cells.

Regulator	Substrate	Ubiquitin chain linkage	Experimental model	Function
E2				
UBC13	BH4-related protein targets	Not fully characterized	Endothelial cell *in vitro*	Regulation of BH4 levels
E3				
CHIP	HSP70/90	Not fully characterized	Bovine aortic endothelial cells	Disrupting the stability of the HSP90-eNOS complex
WWP2	RAP1	K31-linked	HUVECs/WWP2 knockout mouse model	Inhibiting shear- stress-induced ROS production and maintain endothelial barrier integrity
K5	VE-cadherin	Not fully characterized	HHV-8-infected endothelial cells	Inducing endocytosis and degradation of VE-cadherin
CHFR	VE-cadherin	Not fully characterized	HLMECs/CHFR knockout mouse model	Disrupting endothelial integrity
MARCH2	VE-cadherin	Not fully characterized	HUVECs	Downregulating VE-cadherin and compromising endothelial barrier integrity
TRIM40	ROCK1	K48-linked	Type 2 diabetes mouse model	Affect the stability of VE-cadherin-mediated cell junctions
PDZRN3	Junction-associate-d substrates	Not fully characterized	Stroke mouse model/ PDZRN3 knockout mouse model/ HMVECs	Regulating the integrity of inter-endothelial cell junctions
TRAF6	Multiple signaling intermediates	K63-linked	Endotoxemia mouse models/HPMECs/ HUVECs	Mediator of NF-κB signaling pathway activation
TRIM47	TRAF2	K63-linked	TRIM47 knockout mouse model/HUVECs	Activating NF-κB and MAPK signaling and triggering endothelial inflammatory responses
SMURF2	HMGB1	K48-linked	Atherosclerosis mouse model/ HUVECs	Inhibiting vascular endothelial inflammation and macrophage infiltration
MIB1	MAP3K5	Not fully characterized	CMD rat model/cardiac microvascular endothelial cells	Reducing apoptosis and inflammation in cardiac microvascular endothelial cells
UFL1	NLRP3 inflammasome components	Non-degradative	Ufl1 knockout mice/ mouse primary peritoneal macrophages (PMs)	Attenuating pro-inflammatory cytokine release
Parkin	Mitochondrial proteins	K48-linked	Diabetic mouse model/ HUVECs	Relating to mitochondrial dysfunction, excessive ROS and endothelial apoptosis
FBXO32	KLF4	Not fully characterized	Diabetic mice model/mice bone marrow-derived macrophages and mouse monocyte macrophage leukemia cells	Promoting ROS accumulation and endothelial inflammation
MDM2	HIF-1α	K48-linked	HCM mouse models	Decreasing myocardial capillary formation and proangiogenic gene expression
Nedd4-2	ACE2	Not fully characterized	Neurogenic hypertension mouse model/HAECs	Associated with the progression of neurogenic hypertension
MARCH5	PKM2	Not fully characterized	Ischemia-reperfusion injury model	Regulating ferroptosis during myocardial I/R injury
MuRF1	BKCa β1 subunit	K48-linked	Myocardial I/R rat model	Improving coronary vasodilation and coronary perfusion
DUB				
USP40	HSP90β	Not fully characterized	USP40 knockout mouse model/ HUVECs	Protecting pulmonary endothelium from LPS-provoked inflammation and barrier disruption
USP7	PDK1	K48-linked	Acute Lung Injury (ALI) mouse model/ HLMECs	Modulating endothelial inflammatory response and permeability
USP15	Drp1	Not fully characterized	Pulmonary hypertension mouse model	Promoting proliferation of pulmonary artery endothelial cells (PAECs) by regulating mitochondrial dynamics and cell energy metabolism
A20	LUBAC	K63-removal/K48-mediated	A20 knockout mouse embryonic fibroblasts (MEFs).	Down-regulating NF-κB signaling cascade
CYLD	LUBAC	K63-/linear- chain removal	A20 knockout mouse embryonic fibroblasts (MEFs).	Down-regulating NF-κB signaling cascade
OTUD6a	STING	K48-linked	OTUD6a knockout mouse	Suppressing STING-driven inflammatory responses
Others				
K626, K633	VE-cadherin	Not fully characterized	K626 knockout mouse/HUVECs	Associated with elevated vascular permeability

A systematic literature retrieval was conducted across PubMed, Web of Science, and Scopus databases up to September 2026 for English-language articles using combinations of the following terms: “coronary microvascular dysfunction/disease”, “coronary microvascular endothelial cell”, “ubiquitination”, “ubiquitin-proteasome system”, “E3 ubiquitin ligase”, “deubiquitinase”, “eNOS”, “endothelial barrier”, and “endothelial inflammation”. Studies were included if they addressed the ubiquitination pathway in relation to endothelial function, coronary or cardiac microvascular disease, or relevant therapeutic strategies (proteasome inhibitors, E3 ligase and DUB modulators, PROTACs); reviews, original research, and clinical studies were all considered. Reference lists of retrieved articles were screened for additional relevant studies, with emphasis on publications from 2020 to 2026.Relative to earlier reviews of CMD or of the ubiquitin-proteasome system, this review focuses specifically on the mechanistic links between the ubiquitination pathway and coronary microvascular endothelial cell function, integrates recent (2020-2026) findings, discusses contradictory and context-dependent roles of individual E3 ligases and deubiquitinases, and addresses the clinical translation of ubiquitination-based biomarkers and therapeutics for CMD.

## 2 Overview of coronary circulation anatomy and ubiquitination modification

### 2.1 Anatomy and physiological function of coronary circulation

The coronary circulation is a continuum composed of proximal and distal compartments, with a gradually decreasing vascular diameter. Epicardial coronary arteries represent the proximal compartment, with diameters typically exceeding 500 micrometers (*μ*m). They primarily serve a transport function and contribute minimally to physiological resistance, accounting for approximately 5% of total coronary vascular resistance (8). Only when the degree of arterial lumen occlusion exceeds 70% does the stenosis of epicardial arteries have significant hemodynamic significance (9). In contrast, the coronary microcirculation constitutes the distal compartment, serving as a critical network for myocardial blood supply. It has a complex structure, including epicardial pre-arterioles with a diameter of 100–500 μm, arterioles with a diameter of less than 100 μm, capillaries with a diameter of less than 10 μm (Figure 1), and venules with a diameter of 20–50 μm (10). The microcirculatory system accounts for more than 90% of the total coronary vascular bed and serves as the primary determinant of coronary vascular resistance (8). Precapillary resistance vessels (arterioles) regulate blood flow resistance through the contraction and relaxation of vascular smooth muscle cells (VSMCs), and their tone is synergistically modulated by local metabolites (e.g., adenosine, lactic acid), neurotransmitters (norepinephrine), and endothelium-derived factors (NO, endothelin-1) (11). Exchange vessels (capillaries) facilitate the exchange of oxygen and carbon dioxide through tight junctions and pore structures between endothelial cells; meanwhile, post-resistance vessels (venules) participate in the regulation of venous return and myocardial interstitial pressure by modulating vascular compliance (12).

**Figure 1:**
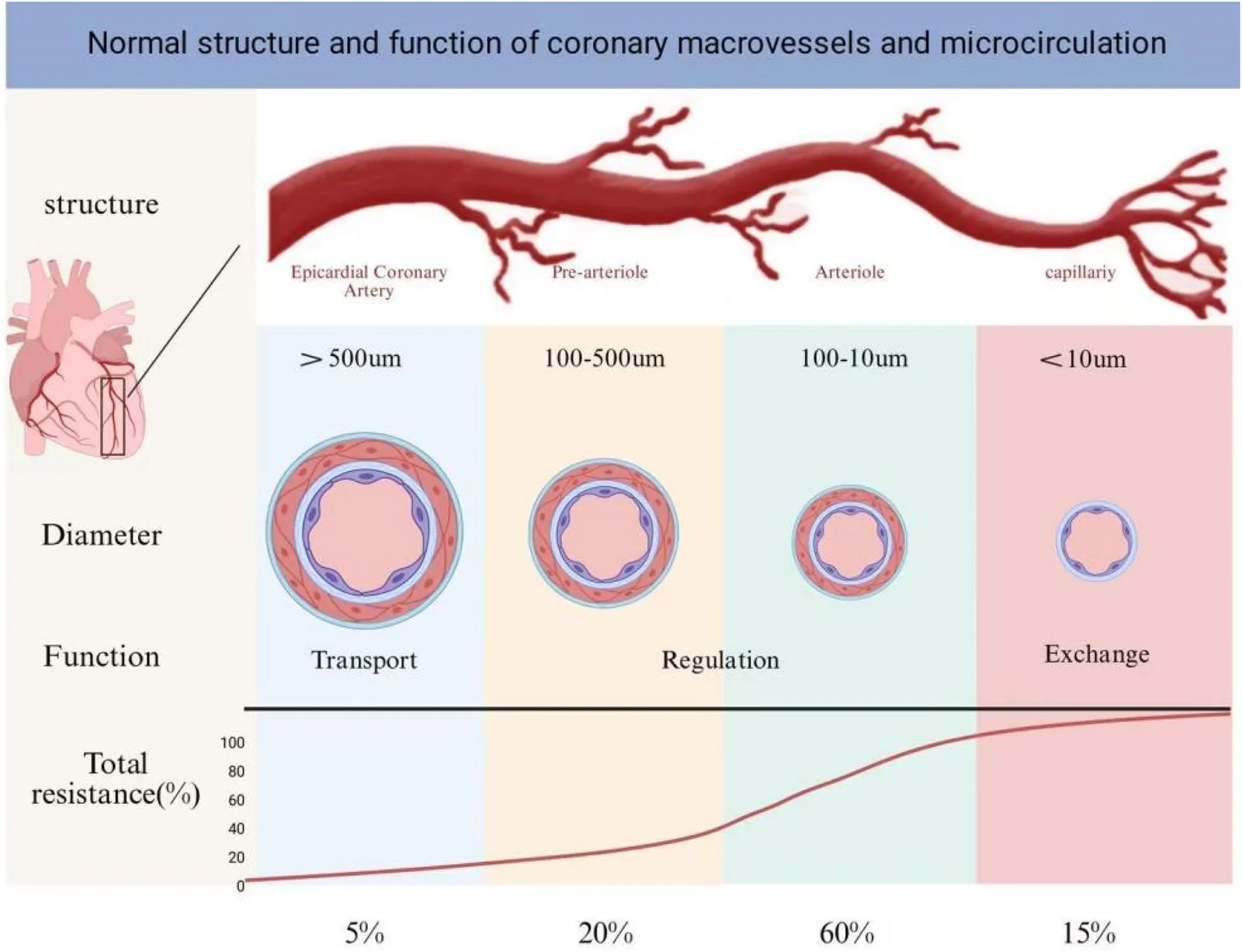
As shown in the figure, the coronary circulatory system consists of proximal and distal compartments. The proximal chamber refers to coronary arteries with a diameter greater than 500 μm, which primarily serve a transport function and account for approximately 5% of the total resistance. The distal chamber refers to the coronary microcirculation with vessels smaller than 500 μm, including pre-arterioles with diameters of 100–500 μm, arterioles with diameters of 10–100 μm, and capillaries with diameters less than 10 μm. The pre-arterioles and arterioles play a regulatory role, while the capillaries are responsible for substance exchange. Together, they account for approximately 95% of the total resistance in the coronary circulation.

When myocardial oxygen consumption increases (e.g., during exercise), local tissue hypoxia activates the mitochondrial ATP-sensitive potassium (KATP) channel, triggering vascular smooth muscle cell (VSMC) hyperpolarization. Hyperpolarization closes voltage-operated calcium channels (VOCs), reduces Ca^2+^ influx, and ultimately induces vasodilation (13). Meanwhile, endothelial cells sense changes in blood flow shear stress, activate endothelial nitric oxide synthase (eNOS) via the phospholipase C-protein kinase C (PLC-PKC) pathway, generate nitric oxide (NO) that diffuses to smooth muscle cells, elevate cyclic guanosine monophosphate (cGMP) levels by activating guanylate cyclase, and ultimately induce myosin light chain dephosphorylation and vasodilation (14). This dual regulatory mechanism ensures that the myocardium receives a stable perfusion of approximately 1.0 mL/min/g at rest while the perfusion volume can increase 4–6 times during strenuous exercise (15).

### 2.2 The molecular mechanism of ubiquitination modification

Ubiquitination, as a highly conserved post-translational modification, is driven by a dynamic enzymatic cascade involving the enzymatic cascade of ubiquitin-activating enzymes (E1), ubiquitin-conjugating enzymes (E2), and ubiquitin ligases (E3), and the counter-regulation by DUBs (16). In humans, this process is initiated by two E1 enzymes—UBA1 and UBA6—which activate the 76-amino-acid ubiquitin molecule in an ATP-dependent manner to form a thioester-linked E1–ubiquitin intermediate (17). Subsequently, the ubiquitin molecule is transferred to the cysteine residue of E2 (approximately 40 types of E2 exist in humans), forming an E2-ubiquitin complex (18). In humans, there are more than 600 types of E3. By recognizing specific motifs of target proteins [e.g., the proline-glutamic acid-serine-threonine (PEST) sequence adjacent to lysine residues], E3 ultimately forms an isopeptide bond between the substrate's lysine residue and the ubiquitin's C-terminus (19) (Figure 2).

**Figure 2:**
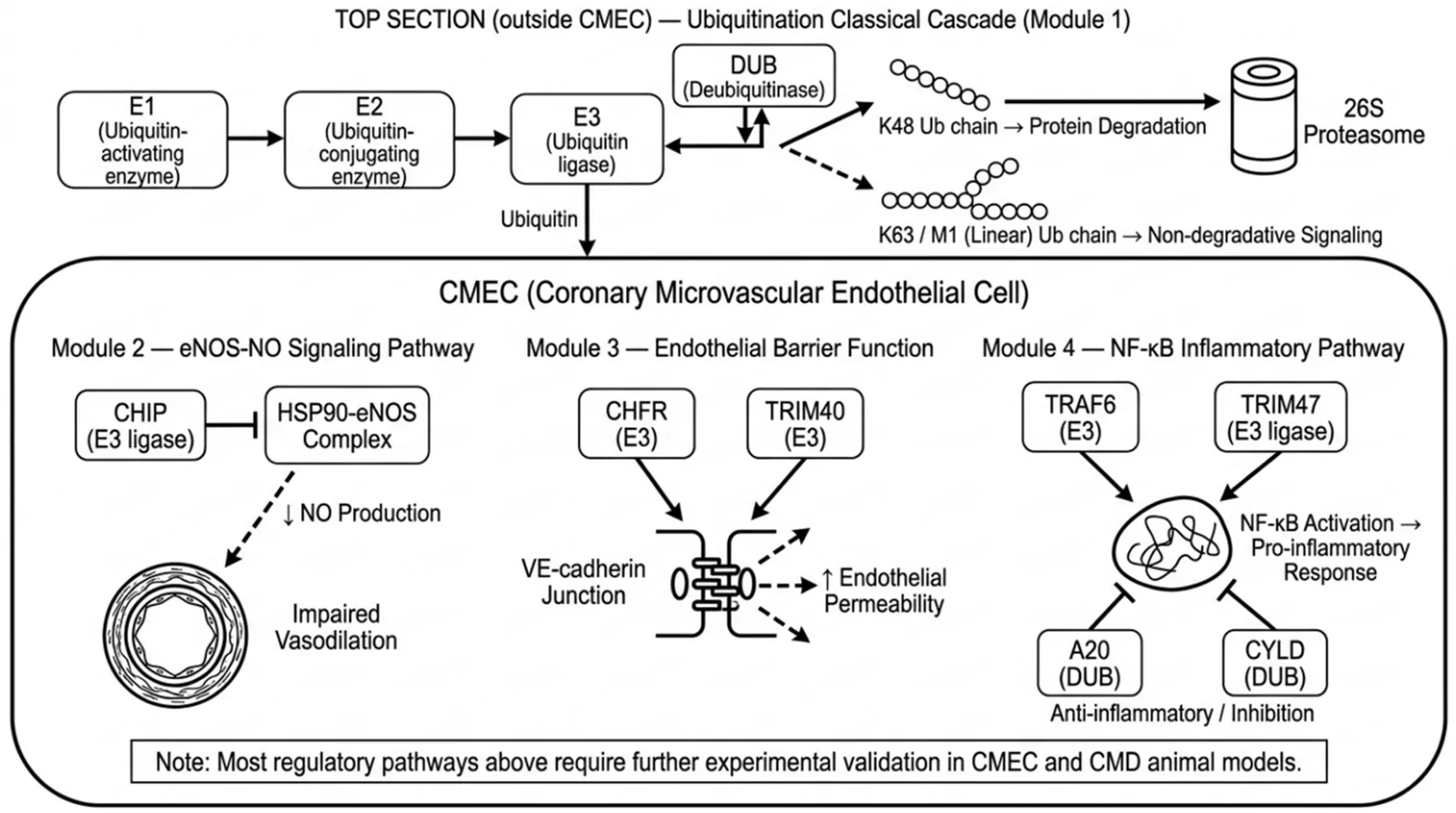
Schematic overview of core ubiquitination-related regulatory networks operating within CMECs. The canonical E1-E2-E3 ubiquitin-ligase cascade and counter-regulatory DUBs govern three major CMECs functional outputs: eNOS-NO signaling, endothelial barrier stability, and NF-*κ*B-driven inflammatory signaling. Representative E3 ligases (CHIP, CHFR, TRAF6, TRIM40, TRIM47) and deubiquitinases (A20, CYLD) discussed in this review are highlighted. K48-linked ubiquitin chains mediate substrate degradation via 26S proteasome, whereas K63-linked or M1-linear ubiquitin chains predominantly transduce non-proteolytic cellular signals. Note that many of these molecular axes await further experimental validation in specific CMD *in-vivo* models.

Depending on the linkage mode and topology of ubiquitin chains, ubiquitination can be classified into multiple categories: K48-linked polyubiquitination typically tags target proteins for degradation via the 26S proteasome (20), whose degradation rate is affected by the length of the ubiquitin chain (the degradation efficiency is significantly improved when there are more than 4 ubiquitin molecules) (21); K63-linked ubiquitin chains do not target proteins for proteasomal degradation but instead play critical roles in various non-proteolytic cellular processes, such as DNA damage repair, cell signaling, and autophagy (22–24); M1-linked linear ubiquitination is catalyzed by the linear ubiquitin chain assembly complex (LUBAC) and plays a key role in inflammasome activation (25). In contrast, monoubiquitination, which commonly occurs at the K120 site of histone H2B, is involved in the regulation of gene transcription (26).

DUBs act as counter-regulatory factors of ubiquitination, removing ubiquitin chains from target proteins by hydrolyzing the isopeptide bond (27). The human genome encodes approximately 100 DUBs, which can be classified into seven families based on their sequence and structural characteristics: ubiquitin-specific proteases (USPs), ubiquitin C-terminal hydrolases (UCHs), ovarian tumor proteases (OTUs), Machado-Joseph disease protease family (MJDs), MINDY proteases (MIU-containing DUB family), JAMM family (JAB1/Pad/MPN domain-containing metalloenzymes), and the newly identified ZUFSP/Mug105 family (zinc finger with UFM1-specific peptidase domain protein/C6orf113/ZUP) (28). Among them, the JAMM family are zinc-dependent metalloproteases, while the other six families are cysteine proteases that exhibit characteristic cysteine residues in the context of a catalytic triad at their active sites (29). Notably, DUBs exhibit high substrate specificity: USP15 can specifically remove ubiquitin chains from the TGF-*β* receptor, thereby maintaining the activity of the Smad signaling pathway (30); while A20 and CYLD inhibit the excessive activation of NF-*κ*B by cleaving the K63-linked ubiquitin chains of TRAF2 (31). This sophisticated regulatory network endows ubiquitination modification with high dynamics and reversibility, making it a key regulatory mechanism for cells to respond to changes in the internal and external environment (32).

## 3 Regulation of coronary microvascular endothelial cell function by ubiquitination

### 3.1 Ubiquitination regulation of the endothelial nitric oxide synthase (eNOS)-NO pathway

Endothelial nitric oxide synthase (eNOS) represents the major source of NO within vascular endothelial cells and is essential for preserving coronary microvascular vasodilatory capacity, whose activity is tightly modulated by post-translational modifications including ubiquitination (33, 34). The stability and catalytic activity of eNOS rely on physical association with the chaperone HSP90. HSP90 interacts with the C-terminal PDZ-containing region of eNOS, stabilizes the conformation of the calmodulin-binding domain, and permits efficient eNOS activation upon Ca^2+^ calmodulin binding (35). The E3 ubiquitin ligase CHIP targets the C-terminal EEVD motif of HSP90. This modification promotes proteasomal degradation of HSP90, dissociates the HSP90-eNOS chaperone complex, and consequently compromises eNOS-dependent NO production (36). Recent studies have also found that mechanical signals can regulate multiple GTPases via non-degradative ubiquitination. For instance, WWP2-mediated ubiquitination of RAP1 promotes the formation of the RAP1/RASIP1 complex, which is critical for maintaining endothelial barrier function (37). These observations indirectly raise the hypothesis that eNOS could undergo analogous non-degradative ubiquitination under mechanical stimuli including flow shear stress. Nevertheless, direct biochemical evidence defining the exact lysine ubiquitination sites and corresponding E3 ligases for eNOS is currently absent.

Furthermore, tetrahydrobiopterin (BH4) is an essential cofactor for the exertion of eNOS function (38). Decreased or oxidized BH4 levels lead to eNOS uncoupling, shifting its product from NO to superoxide (39). Studies have shown that BH4 can regulate the ubiquitin-binding of UBC13/UBE2N as well as proteasomal activity via protein S-nitrosylation (40). This suggests that, influenced by BH4 levels, the biological activity of NO can in turn affect the ubiquitin-proteasome system, and the ubiquitination of eNOS may be indirectly regulated by the BH4-NO signaling pathway. Gallic acid (GA) can inhibit angiotensin II-induced hypertension and vascular dysfunction, and one of its underlying mechanisms is the suppression of eNOS degradation (41). Although that study did not explicitly identify the involvement of ubiquitination, GA has been reported as an immunoproteasome inhibitor in other investigations (42), and the regulation of protein degradation pathways is frequently linked to the ubiquitin-proteasome system (43).

### 3.2 Ubiquitination-mediated regulation of endothelial barrier permeability

Tight junctions and adherens junctions between endothelial cells are key structures that maintain the integrity of the endothelial barrier (44). VE-cadherin is the major component of adherens junctions, whose integrity is essential for endothelial barrier function (45), whereas the ubiquitination modification of junctional proteins plays a dominant role in maintaining the stability of the endothelial barrier (46). Under inflammatory stimulation, the lysine residues K626 and K633 of VE-cadherin undergo ubiquitination, leading to increased endocytosis of VE-cadherin and elevated vascular permeability. Moreover, the ubiquitination of VE-cadherin can also target VE-cadherin to the lysosomal degradation pathway (47). This implies that ubiquitination may promote the degradation or functional alteration of VE-cadherin, thereby impairing the integrity of the endothelial barrier.

Various E3 ubiquitin ligases target VE-cadherin and affect the integrity of endothelial junctions. For example, the ligase K5 encoded by herpesvirus 8 (HHV-8), which exhibits homology to human membrane-associated RING-CH-type ubiquitin ligases (MARCH), can induce the endocytosis and degradation of VE-cadherin (48). Depletion of CHFR in Human Lung Microvascular Endothelial Cells (HLMECs) in culture or specific deletion of the CHFR gene in mice prevented VE-cadherin ubiquitylation and proteasomal degradation (49). In a diabetic mouse model, Rnd3 ameliorates diabetic cardiac microvascular injury by promoting TRIM40-mediated (an E3 ubiquitin ligase) ubiquitination of ROCK1; the underlying mechanism may be that the ubiquitination of ROCK1 affects cytoskeletal remodeling, thereby indirectly modulating the stability of VE-cadherin-mediated cell junctions (50). In addition, in a mouse model of transient ischemic stroke, the E3 ubiquitin ligase PDZ domain-containing ring finger 3 (PDZRN3) regulates vascular permeability by modulating inter-endothelial junctions (51).

### 3.3 Ubiquitination-mediated regulation of endothelial inflammation in coronary microcirculation

Endothelial cells are an important component of the vascular wall, and endothelial inflammation is a critical initiating event under pathological conditions as well as an early indicator of diseases (52, 53). The NF-*κ*B heterodimer is a critical transcription factor that, in its resting state, binds to the inhibitory protein I*κ*B*α* and resides in the cytoplasm (54). Various ubiquitinating enzymes and deubiquitinating enzymes regulate the initiation and progression of endothelial inflammation in coronary microcirculation via the NF-*κ*B signaling pathway (55). E3 ubiquitin ligases play an important role in the initiation and resolution of endothelial inflammation by recognizing specific substrate proteins and mediating their ubiquitination (56, 57).

The E3 ligase TRAF6 (tumor necrosis factor receptor-associated factor 6) mainly generates K63-linked polyubiquitin chains and participates in IL-1β-induced NF-*κ*B activation (58). The Hippo signaling pathway is composed of a serine kinase cascade along with associated regulatory and scaffolding proteins, which transcriptionally regulate the expression of genes that control growth (59). YAP (Yes-associated protein), the major transcriptional coactivator of the Hippo pathway, can regulate the activation of endothelial inflammation and the severity of vascular inflammation (60). In endothelial-specific YAP knockout mice, YAP induces the degradation of TRAF6 via K48-linked ubiquitination, while inhibiting K63-linked autoubiquitination of TRAF6 and suppressing NF-*κ*B signaling, thereby inhibiting vascular endothelial inflammatory responses (61). Additionally, TRIM47, an E3 ubiquitin ligase belonging to the tripartite motif protein family, is widely expressed in multiple tissues and exhibits relatively high expression levels in vascular endothelial cells (62). Studies have shown that TRIM47 enhances K63-linked ubiquitination of TRAF2 to activate the NF-*κ*B and MAPK signaling pathways and trigger inflammatory responses in endothelial cells; these observations are supported by findings from TRIM47-knockout mouse models (63).

Meanwhile, other E3 ubiquitin ligases are also involved in the regulation of endothelial inflammatory responses. For example, SMURF2, an E3 ubiquitin ligase, targets HMGB1 (High Mobility Group box 1 protein) for K48-linked polyubiquitination and subsequent proteasomal degradation. This process modulates endothelial inflammation and monocyte adhesion, thereby attenuating endothelial dysfunction induced by coronary atherosclerosis (64). In a rat model of CMD which is primarily characterized by the apoptosis and inflammation of cardiac microvascular endothelial cells, the overexpression of the E3 ubiquitin ligase MIB1 can reduce the apoptosis and inflammation of these cells (6). This suggests that MIB1 may improve CMD by ubiquitinating mitogen-activated protein kinase kinase kinase (MAP3K5) inhibiting inflammatory pathways, or promoting anti-apoptotic mechanisms. Furthermore, in several NLRP3-associated inflammatory disease models of mice, the E3 ubiquitin ligase UfL1 can prevent K63-linked ubiquitination of the NLRP3 inflammasome, regulate the stability of NLRP3-associated proteins, and attenuate the release of pro-inflammatory cytokines, thereby alleviating inflammatory responses (65).

DUBs counteract or modulate the function of E3 ligases by removing ubiquitination modifications and are also crucial for maintaining homeostasis in endothelial inflammation (66). Ubiquitin-specific protease 40 (USP40) is a newly identified DUB, which mediates the deubiquitination of HSP90*β* and inhibits its activity, thereby protecting pulmonary endothelial cells from LPS-induced inflammatory responses and barrier disruption *in vitro* and in experimental lung injury models, and ultimately maintaining endothelial integrity (67). Studies investigating LPS-induced sepsis-mediated acute lung injury have demonstrated that USP7 promotes endothelial activation via the PDK1/AKT/NF-*κ*B signaling pathway, and inhibiting USP7 can attenuate endothelial inflammatory responses and barrier hyper-permeability (68). Deubiquitinating enzymes such as CYLD and A20 play a negative regulatory role in the activation of IKK (I*κ*B kinase, which is activated by TRAF6) (69, 70). For example, the N-terminal domain of A20, which belongs to the OTU family of DUBs, can remove K63-linked ubiquitin chains from receptor-interacting protein (RIP). Meanwhile, its C-terminal domain, composed of seven Cys2/Cys2 zinc fingers, functions as an E3 ubiquitin ligase that mediates K48-linked polyubiquitination of RIP, thereby targeting RIP for proteasomal degradation. Through these two distinct mechanisms, A20 downregulates the NF-*κ*B signaling pathway (71). Studies have also demonstrated that A20 can inhibit LUBAC-mediated NF-*κ*B activation by binding linear polyubiquitin via its C-terminal seventh zinc finger (ZF7), whereas CYLD suppresses LUBAC-mediated NF-*κ*B activation by hydrolyzing linear ubiquitin chains via its deubiquitinase (DUB) activity (72, 73). CYLD deficiency leads to constitutive NF-*κ*B activation, resulting in the expression of pro-inflammatory genes (74). Through the mechanisms described above, A20 and CYLD collectively attenuate the inflammatory response, which provides insights into the targeted mitigation of inflammation-induced coronary microvascular dysfunction.

### 3.4 Conflicting functions of key E3 ligases and DUBs in vascular biology

Accumulating studies have reported context-dependent, cell-type-specific, and seemingly contradictory biological activities for several well-studied E3 ligases and DUBs related to endothelial function, which complicates direct mechanistic extrapolation to CMD. For instance, as mentioned above, E3 ligases including CHFR, MARCH2, TRIM40 and PDZR3 impair endothelial integrity across different experimental models, whereas USP40 exerts protective effects on endothelial cells. TRAF6 and TRIM47 activate the NF-*κ*B signaling cascade, while A20 and CYLD downregulate it. In a diabetic mouse model, the E3 ligase Parkin is associated with mitochondrial dysfunction, excessive ROS production, and endothelial apoptosis; in a pulmonary-hypertension mouse model, the DUB USP15 promotes the proliferation of pulmonary artery endothelial cells (PAECs) by regulating mitochondrial dynamics and cellular energy metabolism. Notably, multiple mechanistic observations presented in this review are derived from non-cardiac experimental systems, including human umbilical vein endothelial cells (HUVECs), pulmonary endothelial cells, liver-injury models, and pulmonary hypertension models, rather than direct experimental evidence obtained from primary CMECs or *in-vivo* CMD animal models (75). For instance, the protective function of USP40 against endothelial barrier disruption is validated in pulmonary endothelial and acute lung-injury models, and the biological functions of USP15 are mainly characterized in pulmonary hypertension models (75). Despite shared core inflammatory signaling cascades across endothelial subtypes, substantial vascular-bed heterogeneity exists among endothelial populations, including divergent substrate preference for ubiquitin-modifying enzymes and distinct stress-response profiles (76). Therefore, extrapolation of these non-cardiac findings to coronary microvascular pathophysiology requires great caution. Further experiments utilizing CMECs and CMD-relevant *in-vivo* models are essential to validate these regulatory axes within the coronary microcirculation.

## 4 The regulatory role of ubiquitination in CMD induced by other diseases

### 4.1 Ubiquitination regulation of diabetic coronary microcirculatory dysfunction (DCMD)

Multiple studies have shown that diabetes is associated with CMD (77, 78), which is linked to mitochondrial dysfunction, inflammatory responses, oxidative stress, and coronary microcirculatory endothelial cell dysfunction (79). As a key post-translational modification of proteins, ubiquitination may participate in the pathogenesis and progression of DCMD by regulating multiple pathways, including endothelial cell function, inflammatory responses, oxidative stress, and mitochondrial dynamics, under the context of diabetic coronary microcirculation (80, 81). Impaired mitochondria in endothelial cells induce oxidative stress, disrupt metabolism, and activate cellular apoptosis, thereby exacerbating the progression of DCMD (82). Parkin is a cytoplasmic E3 ubiquitin ligase (83). In high-glucose-exposed HUVECs and diabetic mouse models, Parkin knockout attenuates mitophagy, promoting the accumulation of fragmented mitochondria, which leads to mitochondrial dysfunction and excessive ROS production, thereby facilitating endothelial cell apoptosis, exacerbating the progression of DCMD (81, 84). Of note, although the above evidence was derived from different types of ECs, there has not yet been a report on mitophagy in coronary microvascular ECs under hyperglycemic conditions.

In addition, macrophages can induce and sustain pro-inflammatory responses, thereby promoting metabolic and cardiovascular diseases (85, 86). Studies have shown that methylglyoxal (MGO) accumulates significantly in diabetic patients, and the excessive production of small extracellular vesicles (sEVs) derived from MGO-exposed macrophages and reactive oxygen species (ROS) impairs coronary microvascular endothelial function (87, 88). The reason is the substantial accumulation of MGO *in vivo*, which triggers the transcription of the E3 ubiquitin ligase FBXO32. FBXO32 catalyzes the ubiquitination of the transcription factor KLF4, leading to the production and activation of ROS and the induction of endothelial cell inflammation. In the diabetic wound model of mice, FBXO32 knockout can reduce the ubiquitination and degradation of the transcription factor KLF4, enhance the ability of macrophages to phagocytose exogenous apoptotic cells, and alleviate the inflammatory response caused by ROS (89). Alleviating ROS can also inhibit the activation of the NLRP3 inflammasome (90), thereby establishing a favorable positive feedback loop *in vivo*. Under diabetic conditions, Rnd3 can improve diabetic cardiac microvascular injury by promoting Trim40 (an E3 ligase)-mediated ubiquitination of ROCK1, which indirectly regulates VE-cadherin-mediated cell junction stability.

### 4.2 The ubiquitination regulation in CMD induced by hypertension

Under physiological conditions, coronary arteries can regulate their diameter in response to metabolic stimuli, thereby maintaining myocardial oxygen supply (91). However, under hypertensive conditions, this regulatory mechanism is impaired, leading to the onset of CMD (92). The ubiquitination system plays a crucial role in cardiac and vascular pathophysiology, and its dysfunction is closely associated with the development and progression of cardiovascular diseases (93). In the pathological cardiac remodeling induced by hypertension, microvascular dysfunction is prevalent and is considered a significant component in the pathogenesis of hypertrophic cardiomyopathy (HCM) (94). For example, the imbalance of HIF (Hypoxia-Inducible Factor) caused by the E3 ubiquitin ligase MDM2 leads to reduced expression of pro-angiogenic genes during critical periods of myocardial capillary growth, thereby resulting in myocardial hypertrophy (95).

Additionally, endothelial dysfunction induced by hypertension may be associated with abnormal activation or inhibition of the ubiquitination pathway (93). For example, stromal interaction molecule 1 (STIM1) is involved in regulating endothelial function in regulatory T cells (Treg cells) in hypertensive nephropathy (96). It is hypothesized that STIM1 ubiquitination or deubiquitination could modulate Treg-dependent endothelial protection, while no direct experimental evidence in CMD settings has been reported to support this notion. Hypertension is also often accompanied by chronic inflammation and oxidative stress, which impair vascular endothelium and cardiomyocytes (97). Ubiquitin ligases and deubiquitinases can affect the activation of the NF-*κ*B pathway, thereby regulating the production of inflammatory factors (98, 99). For example, hypertension-induced reductions in tissue perfusion and oxygen delivery lead to increased reactive oxygen species (ROS). ROS contributes to oxidative stress, decreases the bioavailability of NO, and cause endothelial dysfunction (100). Angiotensin II is a key mediator of hypertension; it can also induce oxidative stress and inflammation, exacerbating the vicious cycle of vascular injury (92). One study on neurogenic hypertension reported that upregulated expression of the E3 ubiquitin ligase Nedd4-2 is associated with elevated ACE2 ubiquitination. Nedd4-2 may be involved in the ubiquitination of ACE2, thereby contributing to the development of neurogenic hypertension (101). Blockade of ACE2 ubiquitination may attenuate the occurrence and progression of hypertensive coronary microvascular dysfunction.

### 4.3 Ubiquitination regulation of CMD in myocardial ischemia/reperfusion injury (MIRI)

The pathophysiological mechanisms of CMD and MIRI are closely related, and the occurrence and progression of MIRI significantly exacerbate CMD (1). During the process of myocardial ischemia-reperfusion, the excessive production of ROS (oxidative stress) is one of the major driving factors, which serves as a key contributor to the onset of endothelial inflammation (102). MIRI is also a major clinical complication following reperfusion therapy. Its pathogenesis is complex, involving multiple cell death pathways such as oxidative stress, inflammatory responses, apoptosis, necroptosis, ferroptosis, and autophagy (103–106). During ischemia-reperfusion, mitochondria generate excessive ROS, leading to the collapse of mitochondrial membrane potential and the opening of the mitochondrial permeability transition pore (mPTP), which further exacerbates oxidative stress and cell death (107). After reperfusion, inflammatory cells such as neutrophils are recruited to the injured area, releasing large amounts of inflammatory mediators and chemokines, which further exacerbates microvascular damage (108). Patients with CMD are more susceptible to severe injury during ischemic events due to reduced microvascular reserve capacity and inadequate myocardial perfusion. When ischemic myocardium attempts reperfusion, functionally impaired microvasculature fails to effectively restore blood flow, resulting in limited benefits from reperfusion and potentially even more severe damage (109). Studies have demonstrated that the PDCD4 (Programmed cell death 4) gene is significantly upregulated in MIRI (110). In a myocardial I/R injury model, PDCD4 suppresses AKT phosphorylation by inhibiting K63-linked ubiquitination, thereby downregulating BCL-2 expression and exacerbating mitochondrial apoptosis. Thus, knockout of PDCD4 significantly alleviates I/R-induced cardiac injury (111). Additionally, after MIRI, ubiquitination of pyruvate kinase M2 (PKM2) mediated by the E3 ubiquitin ligase March5 is involved in ferroptosis (112). Targeting this pathway may provide a novel therapeutic strategy for acute myocardial infarction.

Calcium-activated potassium channels (KCa channels) are expressed in the coronary microvessels and are closely associated with coronary artery relaxation (113). Large-conductance calcium-activated potassium channels (BKCa) are located on the mitochondrial membrane of adult cardiomyocytes; they increase potassium conductance, enhance mitochondrial respiratory function by reducing ROS production and lowering intramitochondrial calcium levels, and these processes confer cardioprotection against MIRI (114). Wang Sen et al. discovered that chronic intermittent hypobaric hypoxia (CIHH) can enhance BKCa function by reducing inflammatory responses and inhibiting MuRF1 (RING finger protein 1, an E3 ubiquitin ligase)-mediated degradation of the BKCa *β*1 subunit. This promotes coronary artery vasodilation, increases coronary flow perfusion, and thereby protects the heart from ischemia/reperfusion injury (115). Collectively, these findings suggest that chronic intermittent hypobaric hypoxia (CIHH) may exert a protective effect against coronary microcirculation dysfunction (CMD) via boosting BKCa channel function through suppressing MuRF1-dependent degradation of the BKCa *β*1 subunit, which alleviates inflammatory injury and improves coronary microvascular vasodilation and perfusion.

## 5 Targeting the ubiquitination pathway: A novel therapeutic strategy for CMVD

### 5.1 The clinical applications and challenges of proteasome inhibitors

It should be emphasized that therapeutic strategies targeting the ubiquitination pathway for CMD exhibit large heterogeneity in experimental evidence levels. In this section, we explicitly distinguish clinically approved agents, pre-clinical proof-of-concept candidates, and purely theoretical therapeutic targets. Direct *in-vivo* evidence validating most ubiquitin-targeted interventions specifically for CMD remains sparse.

Proteasome inhibitors such as bortezomib and carfilzomib have shown efficacy in the treatment of hematological malignancies (116). By inhibiting the activity of the 26S proteasome, they block the activation of NF-*κ*B and the production of pro-inflammatory cytokines, providing a novel therapeutic approach for the treatment of CMD (117). Low-dose proteasome inhibitors can increase NO production and improve endothelium-dependent vasodilation, an effect that may be related to the inhibition of CHIP-mediated HSP90 ubiquitination (118). Another study showed that carfilzomib reduced TNF*α*-mediated expression of ICAM-1 (Intercellular Adhesion Molecule-1) and TF (Tissue Factor), suggesting that it may exert anti-inflammatory and antithrombotic effects on the endothelium (119). However, the cardiotoxicity of proteasome inhibitors limits their clinical application. A study involving 42 patients with multiple myeloma showed that 23.8% of patients treated with bortezomib experienced cardiovascular adverse events (CAEs) (120). This may be related to the disruption of protein homeostasis caused by proteasome inhibition in cardiomyocytes (121).

Despite promising pre-clinical findings from endothelial cell assays, cardiovascular adverse safety risks severely restrict repurposing proteasome inhibitors for CMD treatment. Bortezomib- and carfilzomib-related cardiotoxicity includes hypertension, heart failure, myocardial ischemia and arrhythmia (122). Toxic phenotypes originate not only from endothelial dysfunction but also disturbed proteostasis inside cardiomyocytes and vascular smooth muscle cells (123). Notably, nearly all available safety data are derived from oncology patient cohorts; dedicated pre-clinical studies exploring low-dose proteasome inhibitors for coronary microvascular protection are scarce. Accordingly, therapeutic translation cannot merely rely on *in-vitro* endothelial observations; rigorous risk-benefit assessment is mandatory for future investigations.

### 5.2 Advancements in the research and development of selective E3 ubiquitin ligase inhibitors

A01, a Smurf1 inhibitor targeting E3 ligases that promote CMD, can competitively bind to the WW domain of Smurf1, inhibit its binding to VE-Cadherin, and reduce endothelial barrier disruption (124). In a mouse model of LPS-induced lung injury, inhibition of the WW domain of Smurf1 reduced vascular permeability by 50%, an effect associated with decreased ubiquitination levels of VE-Cadherin (124). In hypertensive mice with a blocked CD40-TRAF6 signaling pathway, endothelial function was improved and oxidative stress was reduced, suggesting that inhibition of the CD40-TRAF6 signaling pathway in endothelial cells may exert cardioprotective and hypotensive effects (125). The E3 ubiquitin ligase SMURF2 exerts a protective role in atherosclerosis by inhibiting endothelial inflammation, thereby countering disease progression (64). The antiapoptotic E3 ubiquitin ligase (AREL1) plays a role in TGF-*β* induced apoptosis in HUVECs (126); however, its functional relevance in CMD remains speculative and awaits validation in CMEC-based experimental systems.

Although detailed studies on direct vascular endothelial cell-specific E3 ligase inhibitors are currently lacking, it can be inferred that targeting E3 ligases involved in regulating angiogenesis, inflammatory responses, apoptosis, and barrier function in endothelial cells will provide new therapeutic strategies for cardiovascular diseases (CVDs) (56, 57, 127). At present, most E3-ligase-targeting small-molecule compounds have only been evaluated in non-cardiac disease models such as LPS-induced acute lung injury (124). Direct *in-vivo* efficacy data obtained from CMECs-relevant CMD animal models are largely missing. Whether these inhibitors can achieve sufficient target selectivity and adequate drug exposure within coronary microcirculation awaits further experimental validation.

### 5.3 Current research status of DUB modulators

Activating protective DUBs (such as A20, CYLD) or inhibiting pathogenic DUBs (such as USP15) offers new directions for CMD treatment (128, 129). In a mouse model of liver failure, pretreatment with a NOD1 (nucleotide oligomerization domain receptor 1) agonist resulted in significantly higher expression of the anti-apoptotic protein A20. Knockdown of A20 abolished the protective effects of the NOD1 agonist against lipopolysaccharide and D-galactosamine-induced liver injury and hepatocyte apoptosis, whereas its mechanism of action in CMD requires further investigation (130). Moreover, CYLD serves as an important negative regulator of NF-*κ*B via its deubiquitination targets TRAF2, TRAF6, TAK1 (NR2C2) and NEMO, and acts as a key regulator of multiple cellular processes including inflammation (131). Decreased levels of CYLD lead to NF-*κ*B activation and induction of the NLRP3 inflammasome, which holds potential significance for the pathogenesis of inflammatory and autoimmune diseases (132).

Inhibition of DUBs that promote pathological processes has also shown therapeutic potential. In USP15 knockout mice, inhibition of USP15 expression resulted in cell cycle arrest at the G0/G1 phase and a decrease in the proportion of S-phase cells, confirming that USP15 deficiency blocks cell proliferation (133). USP15 stabilizes the TGF-*β* receptor through deubiquitination, promoting the phenotypic switching of vascular smooth muscle cells (VSMCs). Inhibition of USP15 reduces VSMC proliferation and migration. In a rat model of pulmonary arterial hypertension, this inhibition decreases vascular wall thickness and improves microcirculatory perfusion (134). However, the development of DUB modulators faces the challenge of specificity, as most DUBs have a broad substrate spectrum. For instance, in addition to regulating TGF-*β* receptors, USP15 is also involved in DNA damage repair, and non-specific inhibition may lead to genomic instability (135). Recent studies have developed USP15 degraders using PROTAC technology, which degrade USP15 by recruiting the E3 ligase CRBN, achieving specific targeting and demonstrating favorable selectivity in cellular models (136). Similarly, DUB modulators and PROTAC-based degraders for CMD are still confined to early pre-clinical or even purely theoretical stages. To date, no published study has reported *in-vivo* proof-of-concept data demonstrating therapeutic benefits of PROTAC or DUB-targeting molecules specifically for coronary microvascular dysfunction. Major translational barriers include inefficient drug delivery towards cardiac microvascular beds and inherent off-target risks caused by broad substrate spectra for most DUB enzymes (137).

## 6 Challenges and future perspectives in clinical translation

A key interpretative limitation for current knowledge is that a large fraction of endothelial ubiquitination mechanisms are extrapolated from non-coronary endothelial sources. Endothelial heterogeneity across different organs can reshape signal outputs upon inflammatory and oxidative insults. Such heterogeneity imposes constraints when translating results obtained from HUVECs or pulmonary endothelial cells to human CMD pathophysiology. Accordingly, future mechanistic work should prioritize primary CMECs and clinical-relevant CMD animal models to verify these extrapolated regulatory pathways.

In future studies, high-throughput screening and proteomics approaches can be employed to identify key ubiquitinated target proteins and specific E3 ligases that play a role in endothelial barrier dysfunction (138, 139). In addition to the classical E1-E2-E3 cascade reaction, the impact of non-canonical ubiquitination modifications—such as those on N-terminal, cysteine, and serine/threonine residues—on endothelial barrier function is also worthy of in-depth investigation (140). Finally, novel small-molecule inhibitors or activators can be developed to specifically modulate vascular diseases induced by related ubiquitination, thereby restoring endothelial barrier function and improving vascular performance (98). PROTAC molecules consist of an E3 ligase ligand, a target protein ligand, and a linker, which can bring the E3 ligase and target protein into close proximity, thereby promoting ubiquitination and proteasomal degradation of the target protein (141) (Figure 3). In cancer treatment, PROTAC exhibits great therapeutic potential by utilizing the cell's own ubiquitin–proteasome system to specifically degrade pathogenic proteins (142). However, targeting the ubiquitination pathway for the treatment of CMD faces multiple challenges: lack of specificity: The ubiquitination system is involved in a wide range of physiological processes, and non-specific intervention may lead to systemic toxicity, such as cardiotoxicity induced by proteasome inhibitors (142); delivery challenge: PROTACs and DUB modulators are mostly large, membrane-impermeable molecules with unfavorable pharmacokinetics, rapid clearance, and poor tissue selectivity, all of which limit their targeting and delivery efficiency to the microvasculature (143). PROTAC-type degraders display pharmacokinetics, clearance, and distribution that differ substantially from those of conventional small molecules (144), as illustrated by the asialoglycoprotein-receptor-mediated plasma clearance of a heterobifunctional degrader of the cardiovascular target PCSK9 (145). Delivering such degraders specifically to the coronary microcirculation therefore remains a major obstacle; emerging strategies for tissue-selective protein degradation include self-assembling supramolecular targeting chimeras that achieve spatiotemporally resolved degradation *in vivo* (146) and microRNA-responsive nanocarriers for lesion-selective release (147). Dynamic regulatory complexity: Ubiquitination modifications are highly dynamic, and single-target intervention may trigger compensatory pathway activation (148). There is also a lack of clinical biomarkers for directly evaluating ubiquitination pathway activity, making it difficult to monitor therapeutic response. Circulating biomarkers associated with coronary microvascular dysfunction have been examined in clinical cohorts, including a large study of women with angina and no obstructive coronary artery disease in which multiple blood biomarkers were associated with CMD, and recent reviews have summarized both the promise and the current limitations of biomarker-based diagnosis and risk stratification of CMD (149, 150). However, none of these markers specifically reflects ubiquitination status, and dedicated clinical cohort data linking ubiquitination-related markers to CMD diagnosis are still lacking. This represents an important translational gap; future work should establish whether ubiquitinomic profiles or markers of endothelial protein turnover can aid the diagnosis and monitoring of CMD.

**Figure 3:**
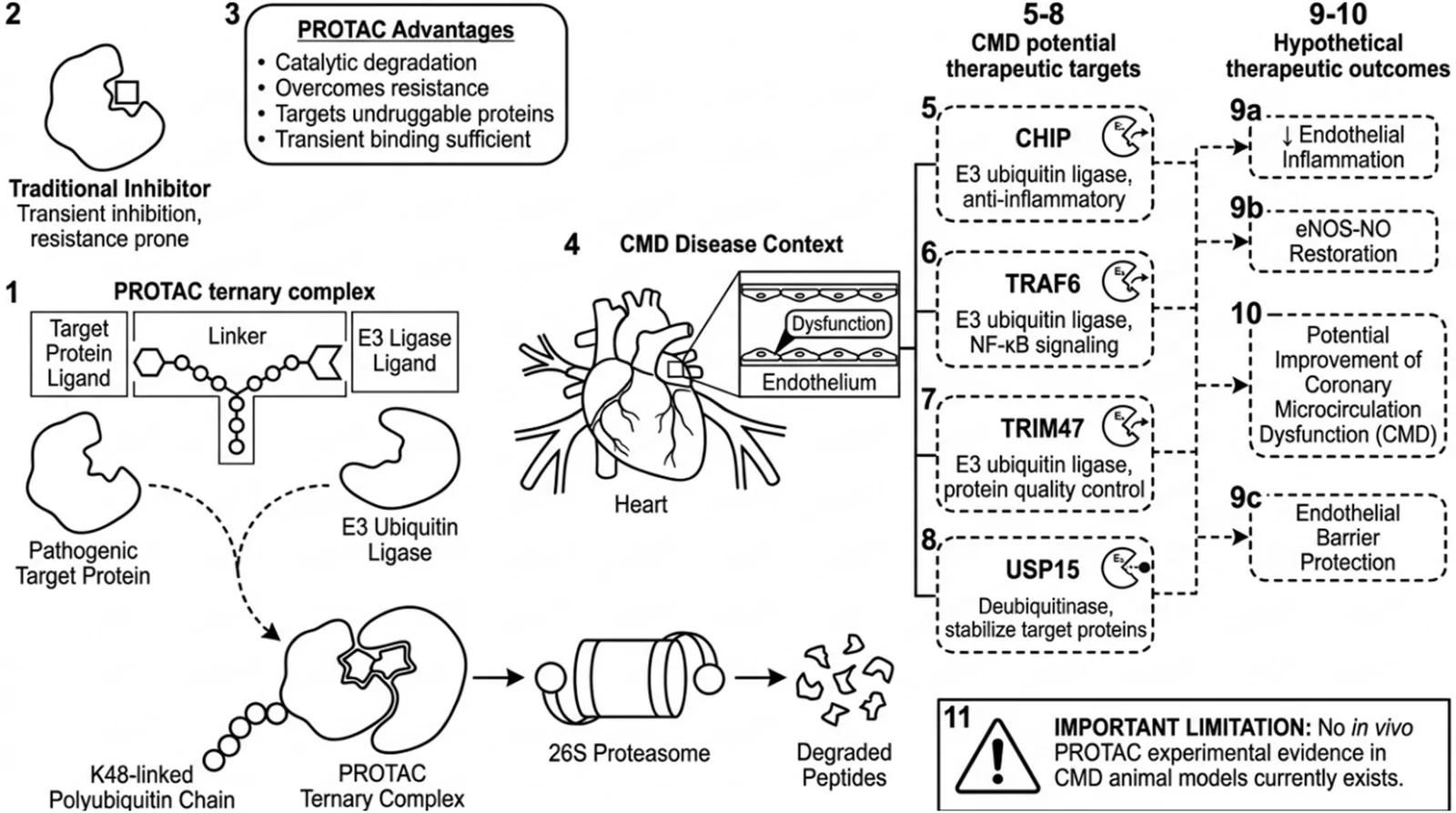
Ubiquitin-mediated regulatory networks in CMECs: schematic overview of core ubiquitination-related regulatory networks operating within CMECs. The canonical E1-E2-E3 ubiquitin-ligase cascade and counter-regulatory DUBs govern three major CMEC functional outputs: eNOS-NO signaling, endothelial barrier stability, and NF-*κ*B-driven inflammatory signaling. Representative E3 ligases (CHIP, CHFR, TRAF6, TRIM40, TRIM47) and deubiquitinases (A20, CYLD) discussed in this review are highlighted. K48-linked ubiquitin chains mediate substrate degradation via 26S proteasome, whereas K63-linked or M1-linear ubiquitin chains predominantly transduce non-proteolytic cellular signals. Note that many of these molecular axes await further experimental validation in specific CMD *in-vivo* models.

Future research on targeting the ubiquitination pathway for CMD therapy should focus on the following directions: ①Develop tissue-specific targeting strategies, such as using microRNA-responsive nanocarriers to achieve selective drug release at CMD lesion sites. ②Combine CRISPR-Cas9 technology to knockout pathogenic E3 ligases in specific cell types, such as endothelial cell-specific knockout of CHIP. ③Explore combination therapy regimens, such as the combined application of proteasome inhibitors and E3 ligase inhibitors, to enhance therapeutic efficacy and reduce drug resistance. ④Establish ubiquitinomic profiling technologies to screen for CMD-associated ubiquitination biomarkers, such as ubiquitinated eNOS fragments in serum, which can serve as biomarkers for endothelial dysfunction.
